# Successful bailout of stent graft stuck in stent occlusion lesion

**DOI:** 10.1007/s12928-020-00695-x

**Published:** 2020-08-25

**Authors:** Tamon Kato, Ayumu Nagae, Takahiro Sakai, Tatsuya Saigusa, Soichiro Ebisawa, Koichiro Kuwahara

**Affiliations:** Department of Cardiovascular Medicine, Shinshu University School of Medicine, 3-1-1 Asahi, Matsumoto, Nagano 390-8621 Japan

VIABAHN^®^ (VIABAHN^®^, W. L. Gore, Flagstaff, AZ, USA) is a safe and effective stent graft for the treatment of peripheral artery disease, especially for very long occlusions and thrombotic lesions [1, 2]. VIABAHN^®^ has an unfolding stored thread delivery system, which carries a risk of imperfections that can catch on a lesion or other structures. Cases of devices becoming stuck before deployment are considered serious events that may need surgical repair.

A 69-year-old man with a 7-year history of hemodialysis for diabetic nephropathy received coronary artery bypass surgery 3 years prior. He first presented at our hospital 2 years ago complaining of right ischemic foot ulceration and right SFA occlusion. We implanted VIABAHN^®^. One year later, he returned with right SFA re-occlusion. We implanted a bare-metal nitinol stent (INNOVA^®^ 7.0 mm × 100 mm, Boston Scientific, MA, USA) in the re-occluded VIABAHN^®^ site. Nine months later, he exhibited yet another right foot ulcer.

**Fig. 1:**
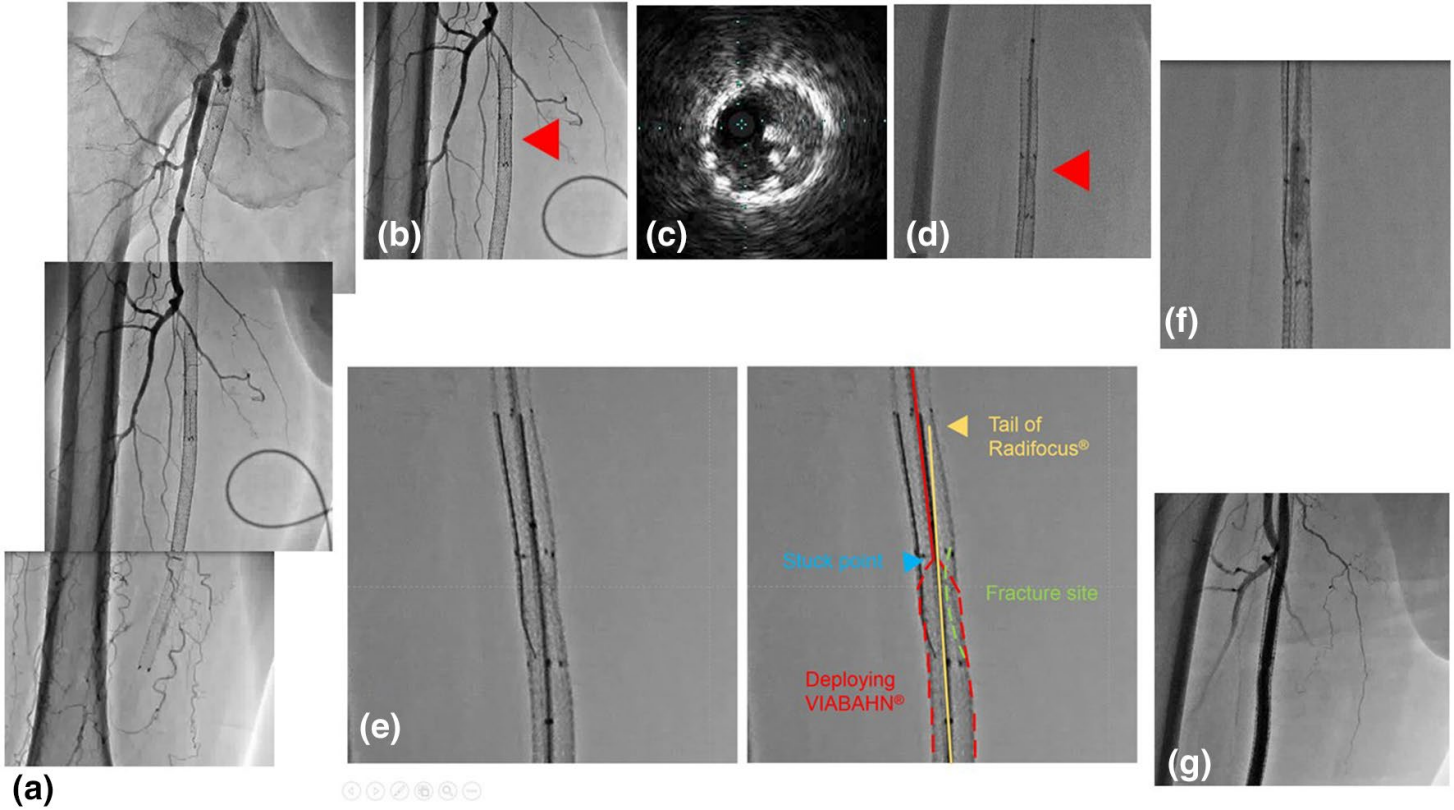
**a** Pre-procedure angiography: re-occlusion from ostium to distal SFA. **b** Stent fracture site (bare metal nitinol stent in VIABAHN^®^ 9 months prior). **c** IVUS findings: stent fracture site. **d** VIABAHN^®^ stuck in stent fracture site. **e** Penetration of stent graft with tail of Radifocus^®^ from retrograde. **f** Ballooning of stuck site. **g** Final angiography

Pre-procedural angiography showed re-occlusion in the right SFA from the ostium to the popliteal artery (Fig. 1a) and a stent fracture site (Fig. 1b) in spite of continued dual antiplatelet therapy (aspirin 100 mg and clopidogrel 75 mg). His ankle–brachial index was 0.57.

We employed a crossover approach from the left common femoral artery with a 6 Fr Destination^®^ (TERUMO Co., Tokyo, Japan). Next, we attempted wiring with a Prominent^®^ microcatheter (TOKAI Medical Products, Aichi, Japan) and Halberd^®^ (ASAHI INTECC CO LTD, Aichi, Japan), with aspiration and distal protection using TOMETAKUN^®^ (ZEON MEDICAL INC, Tokyo, Japan). Intravascular ultrasound (IVUS; Eagle Eye^®^, Volcano Philips, CA, USA) showed a partial stent fracture (Fig. 1c). Repeated balloon angioplasty with a 4.0 mm SABER^®^ (Cordis, Cardinal Health, OH, USA) and aspiration was not effective. We next tried to insert VIABAHN^®^ into the stent. We felt strong resistance during stent placement as the VIABAHN^®^ became stuck at the stent fracture site (Fig. 1d), possibly due to deployment line interference. Antegrade wiring from the brachial artery with a 6 Fr system could not pass the stuck site. Next, we attempted a retrograde approach with a 4 Fr sheath in the popliteal artery. Although neither a Jupiter^®^ 60 DP (Boston Scientific, MA, USA) nor a 0.035 Radifocus^®^ (TERUMO Co., Tokyo, Japan) could be passed, we were able to penetrate a new VIABAHN^®^ with a Radifocus^®^ wire tail from distal to proximal (Fig. 1e). We changed to a Vassallo^®^ (Cordis, Cardinal Health, OH,USA) with NAVICROSS^®^ (TERUMO Co., Tokyo, Japan) and subsequently performed balloon dilatation with a 4 mm × 20 mm Coyote^®^ (Boston Scientific, MA, USA) for the previous fracture stent and stuck point with a newly deployed VIABAHN^®^ (Fig. 1f). IVUS showed that the wire had penetrated the stent graft. After balloon dilatation with a 5.0 mm × 40 mm SABER^®^ (Cordis, Cardinal Health, OH, USA), the resistance disappeared and we could implant the VIABHN^®^ into the previous stent. We completed full coverage of the re-occlusion site and post-ballooning (Fig. 1g).

The retrograde tail of 0.035 wire penetration and balloon dilation are viable options for bailout of VIABAHN^®^ stuck.
